# New Percutaneous Approaches for the Treatment of Heavily Calcified Mitral Valve Stenosis

**DOI:** 10.3390/jcm11216444

**Published:** 2022-10-30

**Authors:** Ricardo Sanz-Ruiz, María Eugenia Vázquez-Álvarez, Enrique Gutiérrez-Ibañes, Felipe Díez-delHoyo, María Tamargo-Delpon, Jorge García-Carreño, Javier Soriano-Trigueros, Jaime Elízaga-Corrales, Francisco Fernández-Avilés

**Affiliations:** 1 Department of Cardiology, Instituto de Investigación Sanitaria Gregorio Marañon (IiSGM), Hospital General Universitario Gregorio Marañón, 28007 Madrid, Spain; 2 Faculty of Medicine, Universidad Complutense de Madrid, 28040 Madrid, Spain; 3 Centro de Investigación Biomédica en Red–Enfermedades Cardiovasculares (CIBERCV), Instituto de Salud Carlos III, 28029 Madrid, Spain

**Keywords:** valvular heart disease, mitral stenosis, percutaneous valve intervention, percutaneous mitral commissurotomy, lithotripsy, mitral annular calcifications, transcatheter valves

## Abstract

Important breakthroughs have considerably improved the outcomes of the percutaneous treatment of valvular heart diseases during the last decades. However, calcium deposition and progressive calcification of the left-sided heart valves present a challenge with prognostic implications that have not been addressed until recently. In the case of native mitral stenosis with no surgical options, a compelling need for tackling heavily calcified valves has led to the development of novel debulking techniques and to the use of aortic balloon-expandable bioprosthesis in the mitral position. In this section of the special issue “Mitral Valve Disease: State of the Art”, we will review standard approaches and indications for the treatment of native mitral stenosis; summarize these two innovative solutions and their evidence, describing both procedures in a “step-by-step” fashion; and briefly comment on future directions in this field.

## 1. Introduction

Structural percutaneous cardiac interventions have revolutionized the treatment of valvular heart disease (VHD) over the last years. Transcatheter aortic valve replacement (TAVR) for aortic stenosis has been extensively investigated and has finally become the standard of care for thousands of patients worldwide, even for those with a low surgical risk [1]. In the case of mitral valve disease, several percutaneous devices have been developed for mitral regurgitation (MR) and more will appear in the near future [2]. With regards to mitral stenosis (MS), percutaneous mitral commissurotomy (PMC) was first described in 1984 by Inoue [3], and the technique itself has experienced very few refinements, although it is still being indicated for an important number of patients [1].

From an epidemiological standpoint, MS is the fourth type of VHD affecting left-sided heart side valves in terms of incidence and is mainly caused by rheumatic fever or degenerative processes. Chest radiation, carcinoid tumor, or atrial mass-derived MS are exceptional. Rheumatic MS is still an important healthcare problem in developing countries, and degenerative MS will increase in the following years in progressively aging developed ones.

## 2. Traditional Percutaneous Approach for Mitral Stenosis

According to the latest clinical practice guidelines, clinically significant (moderate or severe) MS (valve area < 1.5 cm^2^) with symptoms should be treated (class I/level B recommendation), initially with percutaneous dilatation over surgery if no unfavorable characteristics for PMC are present. These include old age, previous commissurotomy, a New York Heart Association (NYHA) functional class IV classification, permanent atrial fibrillation, severe pulmonary hypertension, or prohibitive echocardiographic scores. However, PMC should only be attempted when commissural fusion exists. In other words, this technique is restricted to rheumatic MS (chronic inflammatory disease) and is not useful in degenerative cases (based more in calcium deposits in the valvular and subvalvular apparatus) [1]. However, traditional cardiovascular risk factors and the increasing population age have forced interventional cardiologists to deal with severely calcified valvular anatomies, including mitral annular calcifications (MAC). Indeed, in these same guidelines, a IIa/C recommendation is given to the percutaneous approach in significant symptomatic MS (irrespective of the etiology) without unfavorable characteristics for the PMC. This, together with current clinical practice and real-world scenarios, have, therefore, led to an expansion of the original technique in selected MS degenerative cases. Finally, PMC can be considered in asymptomatic patients with a high risk for thromboembolism or hemodynamic decompensation (IIa/C).

On the other hand, accepted contraindications for PMC include left atrial thrombus, more than mild MR, and severe concomitant aortic/tricuspid disease or coronary artery disease requiring surgery. In theory, severe or bicommissural calcifications (MAC) are also factors that should tip the scale towards valve surgery, but two novel procedures have emerged that may challenge this paradigm in native valves: mitral lithotripsy and “valve-in-MAC” (ViMAC). In the following sections, we will review both techniques, excluding the “valve-in-valve” (ViV) and “valve-in-ring” (ViR) approaches, since they are carried out in non-native (bioprosthetic) mitral valves and rings.

## 3. Lithotripsy-Facilitated Percutaneous Mitral

### 3.1. Commissurotomy

Nowadays, severely calcified aortic valves are well managed with contemporary debulking and specific TAVR devices, a bioprosthesis being immediately delivered (with the exception of aortic valvuloplasty) in most cases. The mitral setting is different, especially for rheumatic MS, in which balloon dilatation is the final treatment (PMC). In cases of heavily calcified mitral valves (rheumatic or degenerative), this “uncontrolled” balloon-based dilatation could lead to undesired tears or ruptures of the calcific tissue in the leaflets (not splitting of the commissures) and severe acute MR that requires emergent surgery. The frequency of this complication ranges from 2% to 19% [4].

Since the 1980s, sonic pressure (shock) waves have been used to fragment renal and biliary stones. After the first in vitro experience by Nowak et al. in 1989 with twelve explanted calcified rheumatic mitral valves, the technology was abandoned due to inefficacy and serious risk of calcium embolism [5]. Thirty years later, and after some surgical experiences, intravascular shockwave systems (Shockwave Medical, Santa Clara, CA, USA) were made available for coronary [6] and peripheral percutaneous interventions [7], and the first case report of lithotripsy-facilitated PMC was published by Eng et al. in 2019 [8]. A patient with severe degenerative MS and an NYHA functional class III classification (considered inoperable) was treated with three peripheral lithotripsy balloons before the final valvular balloon dilatation, which reduced the mean gradient from 11 to 2 mmHg without causing significant MR. Interestingly, the patient was not amenable for ViMAC due to a large valvular area. The risk of embolism was reduced with carotid filters.

One year later, Sharma et al. published another case of lithotripsy-facilitated PMC but in a rheumatic patient in the NYHA functional class IV with concomitant aortic stenosis and severe left ventricular dysfunction [9]. Just after the (longer) shockwave applications, the gradient was significantly reduced (14 to 4 mmHg), but the operators also finished with a balloon dilatation of the valve, not increasing the degree of regurgitation. Embolic protection systems were also used.

Finally, lithotripsy-facilitated PMC has also been performed in a rheumatic setting at the same time as TAVR by our group (“one-step” approach) [10]. A patient with atrial fibrillation, chronic renal failure, severe aortic stenosis, and an NYHA functional class IV classification was turned down by the heart team because of a prohibitive surgical risk. Three lithotripsy balloons prepared the valve before the final mitral balloon dilatation and subsequent TAVR, definitely proving the safety and efficacy of this procedure even when combined with aortic valve interventions, irrespective of the cause of the MS, without cerebral protection devices (not possible due to peripheral radial artery disease).

**Figure 1:**
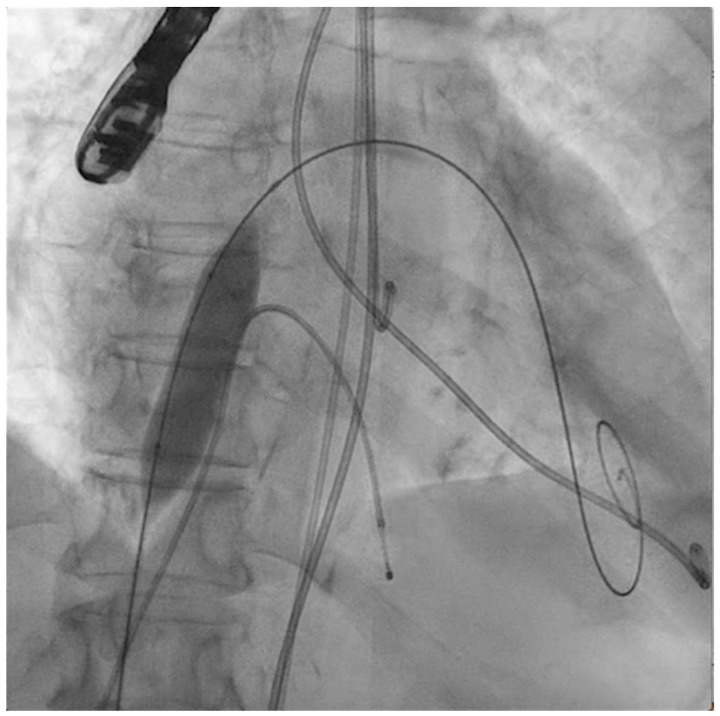
Interatrial septostomy (14 × 40 mm balloon) after transseptal puncture.

The procedure of applying shockwave pulses to the mitral valve is demanding and comes with a learning curve. Given its complexity and duration, general anesthesia is advised. An echography-guided vascular puncture (large-bore sheaths) allows for safe venous and arterial access. At least two femoral veins will be needed (one for transseptal puncture and PMC and the other for temporary right ventricular fast pacing). Transesophageal echocardiography (TEE) guidance is also recommended to attain a mid-posterior transseptal puncture. A high-support wire will retain left ventricular access with the help of a guiding catheter (i.e., multipurpose), which is mandatory to create an interatrial septostomy because of the size of the devices and sheaths to be advanced to the mitral valve (Figure 1).

**Figure 2:**
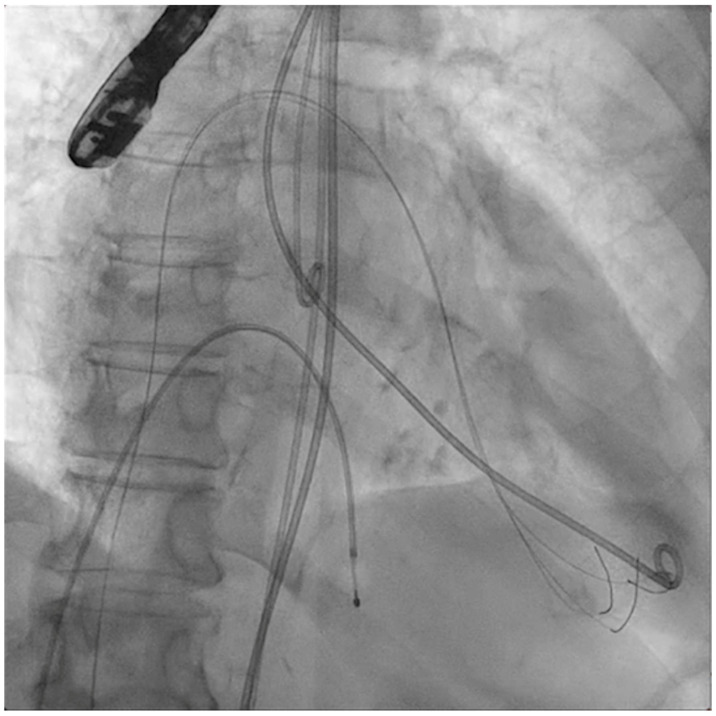
Three angioplasty wires crossing the mitral valve into the left ventricle.

**Figure 3:**
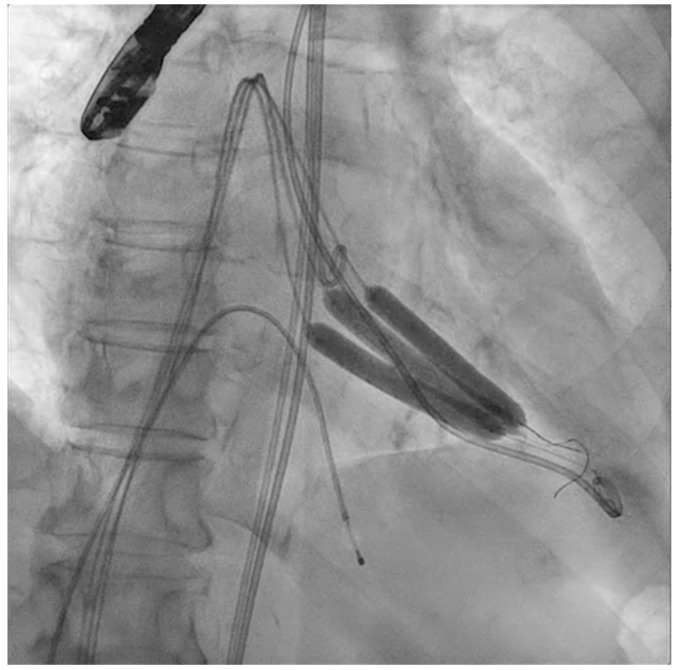
Simultaneous inflation of three 7 × 60 mm shockwave balloons on the mitral valve for lithotripsy applications.

**Figure 4:**
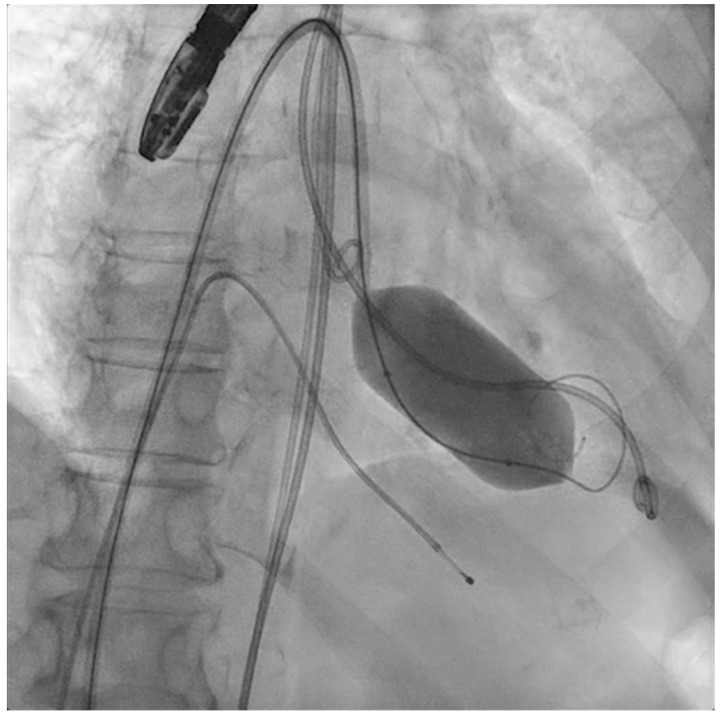
Percutaneous mitral commissurotomy (non-compliant 26 mm balloon).

The next steps consist of crossing the mitral valve with three long high-support 0.014´´ wires downstream into the left ventricle (LV) (Figure 2) and then advancing three lithotripsy balloons (Shockwave Medical) across the mitral valve; with rapid pacing (120 beats/min), simultaneous inflations of all balloons will deliver a total of 90 pulses from each balloon (Figure 3). Finally, access to the LV has to be attained again with the Safari wire to complete PMC with large balloons appropriately sized to the mitral valve area and diameters (Figure 4). Embolic protection devices are encouraged to avoid neurological or systemic ischemic events due to calcific debris.

### 3.2. Valve in Mitral Annular Calcifications (“Valve-in-MAC”)

**Figure 5:**
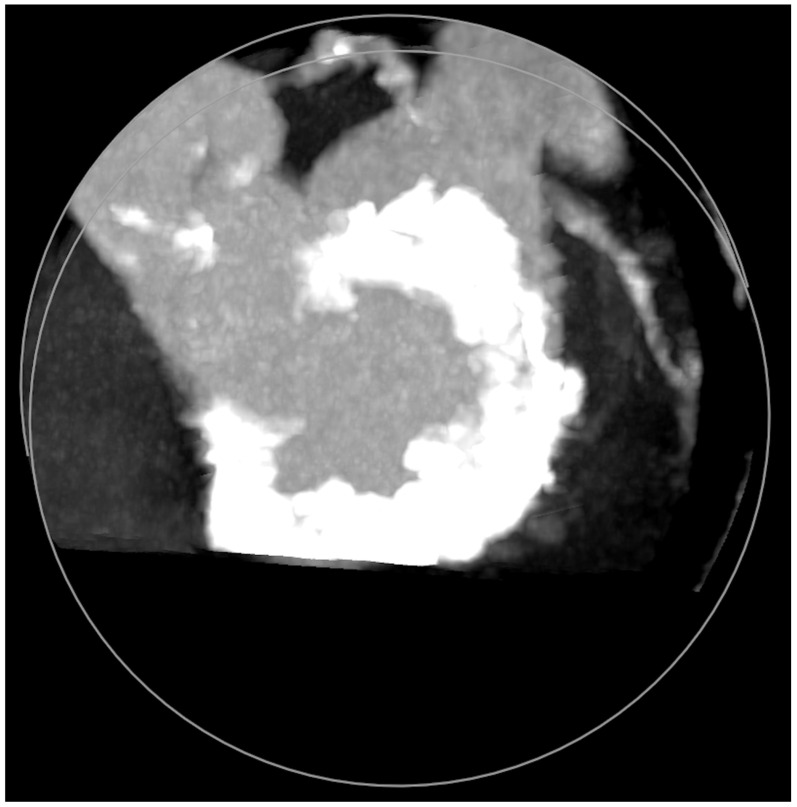
Mitral annular calcifications (MAC), which allow grading of the valvular calcium burden and extension.

MAC consist of circumferential calcific depositions around the mitral valve, especially in the posterior aspect (Figure 5), but sometimes involve the whole mitral fibrotic annulus. It is considered a chronic degenerative condition, although it may be associated with rheumatic mitral disease. It is important to note that MAC has been clearly associated with a worse prognosis due to coronary artery disease and myocardial ischemia, ventricular arrhythmias, cardiovascular events (including mortality), and mitral and aortic valve dysfunctions [11]. MAC more frequently cause MR than MS [12]. Cardiac imaging tools, which are important before mitral lithotripsy, become critical when planning a ViMAC procedure. Apart from transthoracic echocardiography, TEE, and fluoroscopy, cardiac computed tomography (CT) with contrast has emerged as the cornerstone imaging modality since it allows the quantification of the density, severity, and extension of MAC and its relationship to other cardiac structures [13].

Surgical repair or replacement approaches for MAC are challenging, requiring decalcification of the annulus and showing higher rates of periprosthetic paravalvular leaks, “valvular area mismatch” with intra-annular suturing, atrioventricular disruption, stroke, or damage to the left circumflex coronary artery. Other strategies, such as the implantation of valved graft conduits between the left atrium and the LV apex, are less appealing and barely used [13].

With regards to percutaneous solutions and as aforementioned, PMC (with or without lithotripsy) can be attempted in MS with MAC of rheumatic origin, but its efficacy in degenerative MAC is controversial (i.e., absence of commissural fusion). Therefore, new percutaneous or minimally invasive technologies are being developed, taking advantage of the calcific scaffold that lies beneath the mitral valve. These include a variety of balloon-expandable bioprostheses, which can be implanted in MAC in a transseptal, transapical, or transatrial fashion (transcatheter mitral valve replacement: TMVR). The main limitations of this procedure include the following:

(a) Paravalvular leakage (PVL) since the mitral annulus is not circular but D-shaped. This complication can be avoided by prosthesis over-sizing;
(b) Bioprosthesis embolization, in cases of non-complete circumferential MAC;
(c) Obstruction of the LV outflow tract (LVOT) due to displacement of the anterior leaflet of the mitral valve. This event carries higher mortality rates, is more frequent in ViMAC than in ViV or ViR, can be anticipated with precise imaging-based preprocedural planning (CT), and may be solved with midline lacerations of the anterior leaflet (laceration of the anterior mitral leaflet to prevent Outflow obstruction during TMVR: LAMPOON technique) [14] or by means of alcohol septal ablation (ASA) if coronary anatomy is suitable [15];
(d) Similar surgical treatments, valvular mismatch, or the impossibility to implant any available prosthesis in very large mitral annuli.

Some of these complications can be solved with the transatrial (“hybrid”) approach, which requires the multidisciplinary work between cardiac surgeons, anesthesiologists, and interventional cardiologists [16]. Once surgical access to the left atrium has been gained, a balloon-expandable bioprosthesis can be implanted in MAC, and specific surgical techniques may be applied: stitching of the atrial tissue to the prosthesis to avoid PVL and migration, delivering a complete annuloplasty ring above the native annulus in cases of large annuli (>29 mm) before TMVR, excising the anterior mitral leaflet to reduce the risk of LVOT obstruction, and even debriding calcium towards the myocardium, which may perforate the LV.

**Figure 6:**
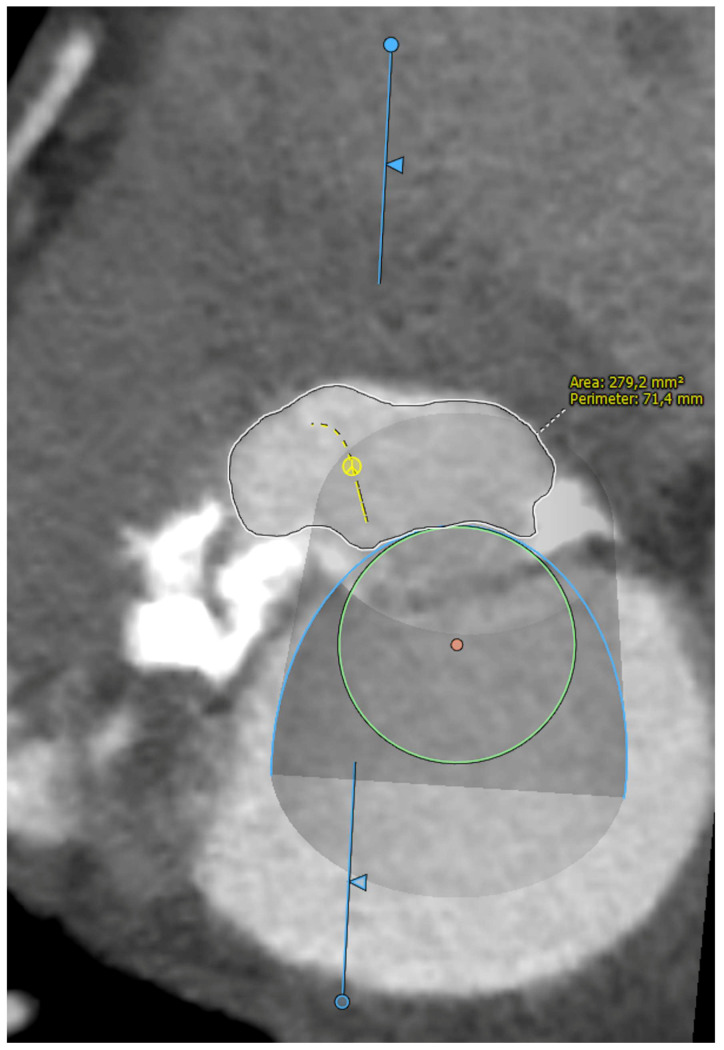
“Virtual” valve reconstruction (blue line) and neo-left ventricular outflow tract measurements.

**Figure 7:**
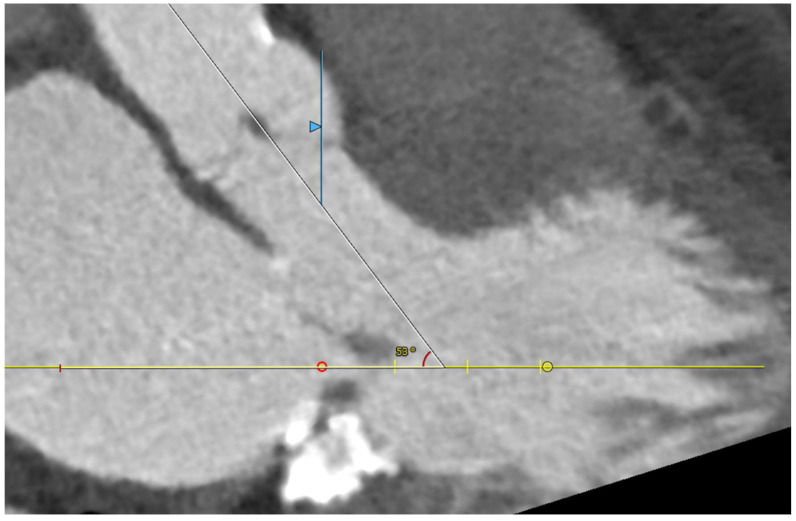
Angle between the aortic and the left ventricular centerlines (aorto-mitral angulation, 53^o^), predictive of left ventricular outflow tract obstruction.

**Figure 8:**
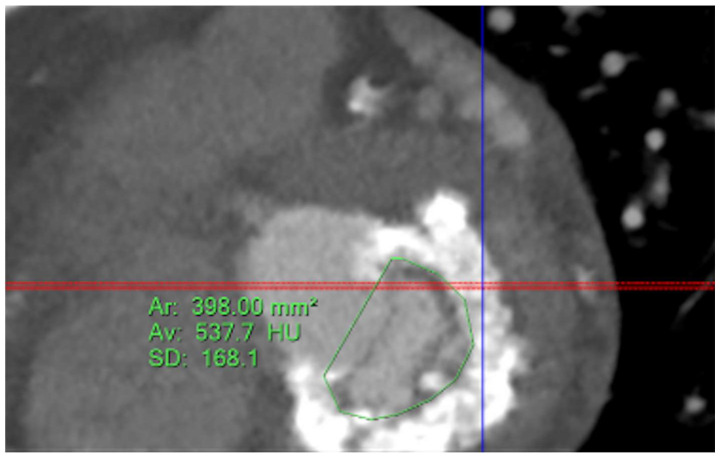
Assessment of mitral annular dimensions and grading of calcium.

Five observational studies have been published since 2017 exploring the safety and efficacy of ViMAC, all of them with aortic balloon-expandable bioprosthesis in prohibitive surgical risk patients [17,18,19,20,21]. Their designs and main results are summarized in Table 1. To note, the ViMAC procedure shows worse results in terms of technical success and short- and mid-term outcomes than those of ViV and ViR. Indeed, ViMAC is the most challenging percutaneous scenario of all types of TMVR. Mortality at the one-year follow-up is high (around 50%), underscoring the importance of patient selection and of performing these interventions in experienced centers. When comparing the three approaches of TMVR for ViMAC, transatrial appears to have the best results, with one-year rates of mortality at 35% (25% in the latest series) [16,22,23], compared to those of transapical (57%) and transseptal (63%), and higher technical success rates (89% vs. 71% and 65%, respectively). The most important adverse event is the obstruction of the LVOT, with a hazard ratio for mortality of 2.87 (95% confidence interval 1.66–4.96) [19], so this must always be anticipated by a preprocedural CT. An area below 170 mm^2^ in the LVOT once the valve is implanted (“neo-LVOT”) is highly predictive of this complication (Figure 6) [13]. Other important CT parameters include the angle between the aorta and the LV axis (Figure 7), mitral annulus dimensions (Figure 8), and 2D- or 3D-derived virtual reconstructions (Figure 6).

From the percutaneous point of view, the procedure is more straightforward than mitral lithotripsy. The material and hardware are very similar to those described above (Mullins sheath, high-support wires, multipurpose or LIMA catheters, non-compliant balloons for septostomy, etc.), but in this setting, mitral pre-dilatation is rarely required (around 10–15% of the cases). Once transseptal access is attained (preferably with TEE guidance) via the femoral vein, interatrial septostomy is needed to ease the advancement of the valve delivery systems. When the stiff wire is positioned in the LV, it can be externalized as a “loop” through a transapical access in difficult cases (“apical rail”). Furthermore, under rapid pacing, the aortic valve will be delivered and implanted according to standard techniques. Newer generation balloon-expandable valves with smaller sheath profiles and outer skirts (i.e., SAPIEN 3, Edwards Lifesciences, Irvine, CA) may improve navigation and annular sealing, thus reducing paravalvular leaks. The valve size and type will be determined by a CT, based on mitral annular dimensions and LVOT measurements. If neo-LVOT (after valve implantation) is < 220 mm^2^ or anterior mitral leaflet length is ≥ LVOT width on the CT, pre-emptive ASA or LAMPOON should be performed. It is important to note that the direction in which the valve is mounted on the balloon (if transseptal) must be the contrary to the one for TAVR. Once the procedure is finished, an atrial septal defect closure may be needed in 30–50% of the patients to avoid left-to-right shunts. Cerebrovascular protection devices are also advised in ViMAC.

## 4. Future Directions

Shockwave-based lithotripsy has more applications in addition to the treatment of MS. For instance, it has also been proved safe and effective when dealing with calcified mitral valves with severe regurgitation to aid in transcatheter edge-to-edge repair procedures. In a case report by Fam et al., calcific deposits in the posterior leaflet/annulus had to be debulked with two lithotripsy balloons before PMC and finally edge-to-edge repaired with a MitraClip (Abbott Vascular, Abbott Park, IL, USA) [24]. Furthermore, although newer TAVR iterations can successfully treat calcified aortic valves without previous lithotripsy, shockwave may be used for extremely calcified aortic valves before valve implantation to improve expansion/apposition and outcomes.

In the MAC-derived MS setting, two studies are still in the recruiting phase to further explore the clinical outcomes of TMVR and/or mitral preparation strategies before TMVR:

(a) In MITRAL II (NCT04408430), a prospective non-randomized multicenter trial, 110 patients will be treated with ViMAC (SAPIEN 3 and SAPIEN 3 Ultra), and 100 patients not suitable for ViMAC will be treated with optimal medical treatment and will serve as controls.
(b) In SITRAL (NCT02830204), 30 patients at a high risk for mitral valve surgery or deemed inoperable due to the extent of calcification will be treated with surgical SAPIEN 3 valve implantation.

Finally, novel minimally invasive (surgical) valvular technologies have been designed for MAC with MR and have already showed promising results, such as the NeoChord (artificial chordae delivery system; NeoChord, Louis Park, MN, USA) and the Tendyne (transcatheter bioprosthetic valve; Abbott Structural, Santa Clara, CA, USA) devices [13]. Other valves with ongoing trials include the following: Intrepid (Medtronic, Minneapolis, MN, USA) in the APOLLO trial (NCT03242642), Half Moon (Half Moon Medical, The Foundry, Menlo Park, CA, USA) in their early feasibility study (NCT04343313), AltaValve (4C Medical Technologies, Maple Grove, MN, USA) in a pivotal trial (NCT03997305), and EVOQUE Eos mitral valve replacement system (Edwards Lifesciences, Irvine, CA, USA) in a safety trial (NCT02718001). Although primarily designed for MR, in the next years, these devices will be probably used for MS in MAC.

## 5. Conclusions

Calcific deposits stand as one of the strongest enemies for interventional cardiologists when treating coronary arteries and also valvular structures. Calcifications are linked with worse outcomes and entail a grim prognosis in different forms of cardiovascular disease. Given the unstoppable and progressive aging of populations worldwide, more patients will require calcium-modifying technologies in the future. Currently restricted to inoperable, high/extreme surgical risk, and no-option patients, these technologies will broaden their indications in the next years, as it has happened with TAVR.

In the mitral valve scenario, good results have been demonstrated with shockwave lithotripsy balloons in case reports of MS with a large calcium burden. Disrupting and debulking just calcific tissue may offer better results after PMC without damaging mitral leaflets or subvalvular structures. Despite the absence of prospective studies and randomized clinical trials, this approach will expand in the following years as per clinical indications in degenerative and especially in rheumatic MS.

Regarding TMVR for MS, the best results have been reported for ViV procedures, followed by ViR. ViMAC remains the most challenging setting, but with new valve developments, multimodality imaging tools, better supporting technologies, and critical analysis of the results of ongoing studies, percutaneous and hybrid treatments for MAC will improve their outcomes with lower and acceptable mortality rates in the years to come.

Thus, either by mitral lithotripsy before valve dilatation or by bioprosthetic valve implantation in severely calcified native mitral annuli, we will be able to offer optimized percutaneous solutions and, eventually, a better prognosis to selected patients with MS.

## Figures and Tables

**Table 1:** Main observational studies of transcatheter mitral valve replacement for mitral stenosis, with a focus on valve-in-mitral annular calcifications cases.

Study	*n*	Design	Type of Valve	Transseptal Approach *	Technical Success **	Outcome	Follow-up	Results
Eleid 2017 [17]	12 (ViMAC arm)	Retrospective multicenter registry	Edwards SAPIEN (100%)	97%	75%	Death or cardiac surgery	30 days−1 year	22−32%
Guerrero 2018 [18]	106	Retrospective multicenter registry	Edwards SAPIEN (98%), Inovare (2%)	41%	77%	All-cause mortality	3 months−1 year	25−54%
Yoon 2019 [19]	58 (ViMAC arm)	Prospective multicenter registry	Edwards SAPIEN (81%), Lotus (16%), Direct Flow (3%)	53%	62%	All-cause mortality	3 months−1 year	35−63%
Tiwana 2020 [20]	28 (ViMAC arm)	Retrospective single-center registry	Edwards SAPIEN (100%)	86%	57%	All-cause mortality	30 days	21%
Eng 2021 [21]	19 (ViMAC arm)	Prospective single-center registry	Edwards SAPIEN (100%)	95%	63%	All-cause mortality	1 year	58%

ViMAC: valve-in-mitral annular calcifications. * remaining approaches included transapical or transatrial. ** according to Mitral Valve Academic Research Consortium (MVARC) criteria.

## Data Availability

Not applicable.
